# A Logical Framework for Forensic DNA Interpretation

**DOI:** 10.3390/genes13060957

**Published:** 2022-05-27

**Authors:** Tacha Hicks, John Buckleton, Vincent Castella, Ian Evett, Graham Jackson

**Affiliations:** 1 Forensic Genetics Unit, University Center of Legal Medicine, Lausanne—Geneva, Lausanne University Hospital and University of Lausanne, 1000 Lausanne 25, Switzerland; vincent.castella@chuv.ch; 2 Fondation pour la Formation Continue Universitaire Lausannoise (UNIL-EPFL) & School of Criminal Justice, Batochime, 1015 Lausanne, Switzerland; 3 Department of Statistics, University of Auckland, Private Bag 92019, Auckland 1142, New Zealand; john.buckleton@esr.cri.nz; 4 Institute of Environmental Science and Research Limited, Private Bag 92021, Auckland 1142, New Zealand; 5 Principal Forensic Services Ltd., Bromley BR1 2EB, UK; ianevett@btinternet.com; 6 Advance Forensic Science, St. Andrews KY16 0NA, UK; advanceforensicscience@gmail.com; 7 School of Applied Sciences, Division of Psychology and Forensic Science, Abertay University, Bell Street, Dundee DD1 1HG, UK

**Keywords:** DNA, forensic, principles of interpretation, investigative, evaluative, reporting, LR, propositions, activity issues, transfer

## Abstract

The forensic community has devoted much effort over the last decades to the development of a logical framework for forensic interpretation, which is essential for the safe administration of justice. We review the research and guidelines that have been published and provide examples of how to implement them in casework. After a discussion on uncertainty in the criminal trial and the roles that the DNA scientist may take, we present the principles of interpretation for evaluative reporting. We show how their application helps to avoid a common fallacy and present strategies that DNA scientists can apply so that they do not transpose the conditional. We then discuss the hierarchy of propositions and explain why it is considered a fundamental concept for the evaluation of biological results and the differences between assessing results given propositions that are at the source level or the activity level. We show the importance of pre-assessment, especially when the questions relate to the alleged activities, and when transfer and persistence need to be considered by the scientists to guide the court. We conclude with a discussion on statement writing and testimony. This provides guidance on how DNA scientists can report in a balanced, transparent, and logical way.

## 1. Introduction

In this article we discuss a framework that has been established by forensic scientists (and by extension, forensic DNA scientists) to help them reason about, and convey, their findings in a balanced, robust, logical, and transparent way. This form of reasoning was applied as early as the end of the 19th century [1]. It was formalised by the Case Assessment and Interpretation team of the former Forensic Science Service of England and Wales in the 1990’s [2]. The approach is a paradigm for reasoning in the face of uncertainty, whatever the forensic discipline, although here we focus on DNA. This paradigm, which we consider to be the fundamental basis for reasoning in all forensic science disciplines, is discussed in numerous books [3,4,5,6,7,8] and interpretation guidelines [9,10,11,12].

## 2. Uncertainty in the Criminal Trial

In the context of a criminal trial, there are few elements that are known unequivocally to be true: the court is uncertain about key disputed events but needs to give a verdict. Disputed events could be, for example, whether “Mr Smith is the father of the child”, or if “Ms Jones is the source of the blood that has been recovered from the crime scene”. In those cases, the court will seek the help of DNA scientists. Very often, the court will have high expectations and expect definitive answers. Because of the CSI effect [13], they might believe that DNA is unique and, as such, will allow the identification of the person who is the father of the child or is the origin of the blood/DNA found at a crime scene. However, things are not that simple: the approach that is used does not allow the formal identification of the person who is the father of the child or who is the origin of the trace. There are very few forensic results that can be presented as “facts” and where one can be categorical. Is this problematic? We argue that it is not: uncertainty exists and, as explained by Dennis Lindley [14], rather than neglecting it or wanting to suppress it, the best approach is to find a logical way to manage it. As we will see, this is done by invoking the notion of probability, which provides a coherent logical basis for reasoning in the face of uncertainty.

## 3. Roles of the DNA Scientist and Different Type of Reporting (Factual, Investigative, Evaluative)

Information given by DNA scientists may be factual or may be in the form of an opinion. (Here, we refer to opinions based on knowledge and professional judgement (i.e., inferences drawn on the basis of forensic observations)).

A factual report describes what has been done and the observations obtained. The scientist makes no inference based on these observations and offers no opinion on the meaning of the results. Factual reporting is appropriate when conclusions are straightforward. A typical example would be a report where the DNA profile of a person is described so that it can be entered into a national DNA database. If expert knowledge is needed to draw a conclusion from the observations, then it would be misleading to present only the observations without offering a professional opinion. An example would be if the scientist only reported the description of the results of a presumptive test for blood. Simply stating that the item was tested for the presence of blood and that the result was positive could be misleading. Indeed, one cannot assume that a positive presumptive test for blood demonstrates unequivocally that the material is blood, even in the investigation stage [15]. Similarly, reporting in a paternity case that the child and the alleged father share one allele in common for all but one locus, without offering an opinion, could be easily misunderstood.

It has been suggested that the opinions given by forensic scientists can be classified broadly into two types—"investigative” and “evaluative” [16]. This should not be taken to mean that forensic scientists conduct police investigation or that they work as investigators. The point of this distinction is to underline that the questions encountered during the investigation and the court proceedings generally are of a different form. This leads to a difference in the inferential process [16,17] used in the generation of the opinion that then contributes to addressing these questions. Examples of the different activities that typify the two different roles that DNA scientists can take is shown in Table 1. It must be stressed that sometimes it may be difficult to separate these roles unequivocally.

An investigative opinion arises when explanations are generated to account for the observations. They are generally, but not exclusively, made in the absence of a person of interest (POI). An investigative opinion could be given in a case where a victim of a possible rape does not have a clear recollection of what happened and where no semen is recovered. Possible explanations for the absence of sperm could be that a condom was worn, or that there was no ejaculation or that all trace of sperm was lost, or that the victim used a vaginal douche or that there was no sexual intercourse. The list of explanations offered by the scientist may not be exhaustive—there may be other possible explanations that the scientist has not considered or has not been able to generate, and they are not necessarily mutually exclusive (i.e., several explanations might be true [18], for example maybe a condom was worn and the victim used a vaginal douche). Another example of an investigative opinion could be a case where a DNA analysis of an athlete’s urine is performed in the context of possible doping. Imagine that the single DNA profile derived from the urine does not align with the DNA profile of the athlete, but that many alleles are shared between both DNA profiles. In such a case, provided no error has been made, the athlete cannot be the source of the urine. A possible explanation for the findings would be that a close relative is the source of the urine. This could be suggested as a possible avenue of investigation.

As mentioned, the separation of the roles and the types of opinion provided by forensic scientists is not always straightforward. There is nothing wrong in assessing the value of a DNA comparison for investigative purposes, for example. This is typically the case where a database search is carried out, when there is no suspect associated with the scene. Here, the aim is to provide investigative leads and information on who could be the source of the DNA. The main difference is that in the initial stages of a case, there might be no suspect/defendant. When there is, if the results are meant to be used in court, the scientist will need to take into account at least one alternative, for example, the defence’s perspective of events. This may be based on what the defendant says, but as there is no obligation for the defence to provide information, this can also be grounded in case information gathered during the investigation (e.g., the defendant works at the same place as the victim) and/or in what appears to be a reasonable alternative (i.e., a proposition that would be amenable to a reasoned assignment of credibility by a judicial body). In a case in which there is a defendant, scientists should offer an evaluative opinion on their results, based upon a pair of case-specific propositions (sometimes also called “allegations” or “hypotheses”) and the framework of circumstances. According to Willis et al. [9], evaluative reports for use in court should be produced when two conditions are met:

1. The forensic practitioner has been asked by a mandating authority or party to examine and/or compare material (typically recovered trace material with reference material from known potential sources).
2. The forensic practitioner seeks to evaluate the results with respect to particular competing propositions set by the specific case circumstances or as indicated by the mandating authority.

## 4. Desiderata and Principles of Interpretation for Evaluative Reporting

When choosing the approach for the evaluation of forensic findings, there is a need to first define what the desired properties of the interpretation framework are [16,19]. The desiderata of any approach to interpretation have been proposed as: balance, logic, transparency, and robustness. Not unsurprisingly, these have since been included in several guidelines on evaluative reporting [9,10,12,20]. This is not to say that investigative opinions do not have these properties. However, the principles of interpretation [4,21,22] apply mainly for forming evaluative opinions. This is because in the early stages of an investigation, there may be very little case information, little in the way of suggestions for what happened, and no suspect. In that case, it would not be possible to consider alternatives put forward by, for example, a defence team. Below, we consider only the principles for evaluative reporting as applied to issues relating to a POI when their case proceeds to court.

### 4.1. First Principle of Evaluative Reporting: Importance of Case Information

The first principle for forming evaluative opinions tells us that interpretation takes place in a framework of circumstances. Note that this is also true for our choice regarding the methods of analysis: we would need to know what the issue is. One can distinguish between aspects of the circumstances that are task-pertinent and task-irrelevant. The role of the forensic scientist can be divided in two parts: (1) understanding the uncertainties facing the fact finder and (2) helping the fact finder to resolve them. They will thus first need information to identify the issue(s) with which forensic science can help. Then, the scientist should devise and agree on an effective case strategy, including an assessment of the possible outcomes and their value, to help address the issue. Once the examinations have been completed and observations have been made, scientists will assign a probability to the specific observations, given propositions that are meaningful in the case and given the available information (task-pertinent case circumstances, but also expert knowledge). The probability they assign will be personal and conditional in the sense that it depends on what the individual knows, is told, and what is assumed. For a DNA scientist, case circumstances such as whether the persons of interest have legitimate access to the objects/persons/premises, what they say about the alleged incident in question, the activities that are alleged to have taken place, and the timelines, are all examples of task-pertinent information that will impact the value of the results. Examples of information that is not relevant for the DNA scientist and is potentially harmful because of bias could include (1) there is eyewitness evidence that points toward the POI, (2) there is a partial finger-mark that supports the proposition that a specified person touched the object, or (3) the POI had first confessed to the offence but later retracted that admission. As task-pertinent case information impacts the value of the findings, it is essential to give a signal that should the framework of circumstances change, it will be necessary to review the interpretation. We discuss examples of caveats in Section 12.

### 4.2. Second Principle of Evaluative Reporting: Two or More Competing Propositions Should Be Considered

To be balanced, when assessing the value of biological results, one must consider at least two propositions (i.e., statements that are either true or false, and that can be affirmed or denied). They will be formulated in pairs based on the case information (we discuss proposition formulation in Section 7) and should represent the views of the two parties as understood at the time of the writing of the report.

Propositions need to be mutually exclusive (if one is true, the other is not) and, if possible, exhaustive in the context of the case (i.e., one should not consider all propositions as default, but only those that are of interest to the court [23]). It is not feasible nor desirable to consider absolute exhaustiveness, and practice can proceed with an acceptable coverage, that is without the omission of a relevant proposition [24]. It is important to ensure that the propositions to be considered are based on case information. If, the case information changes and it is shown that the propositions considered are not meaningful anymore, a new evaluation will need to be performed and a new written statement issued.

### 4.3. Third Principle of Evaluative Reporting: Scientists Need to Assign Their Probability of the Findings, Not Their Probability of the (Alleged) Facts

To respect logic, forensic scientists shall assign the probability of their findings given the truth of propositions, not the probability of the propositions given the findings. It may seem obvious that scientists need to focus on the value of the findings. However, it is not a straightforward endeavour, and many scientists are tempted to give an opinion on propositions. An example could be the doping case discussed earlier: while the DNA scientist is in a position to assign the probability of the results given the urine is from the athlete’s sister, it is not possible—based on the results only—to assign the probability that the urine is from the athlete’s sister. To do so, one would need to consider all the information in the case (for example that the athlete has a sister).

### 4.4. Forth Principle of Evaluative Reporting: The Value of the Findings Is Expressed by the Ratio of the Probability of the Scientific Observations Given the Case Information and Given That Each, in Turn, of the Propositions Are True

To measure how the new evidence (e.g., DNA results) affects one’s uncertainty about the proposition (e.g., the urine is from the sister) considering conditioning information, one can use a model which is known as Bayes’ rule. It is a mathematical idealisation that the belief about a set of propositions is updated based on the (weight of the) evidence [25]. Note that Bayes’ rule is seldom used in court (except maybe for paternity cases). Generally, the different pieces of evidence are combined intuitively by the fact finder without assigning any figure. This is done, for instance, when referring to “corroborating evidence”. Notwithstanding, Bayes’ rule provides a very useful framework for understanding how DNA results may be presented in a logical, transparent, and impartial way in legal proceedings.

Bayes’ rule may be depicted in a format known as the odds form of Bayes’ rule (Equation (1)). Odds are the ratio of the probability of the proposition being true divided by the probability of it being false.

(1) $$\underset{\mathrm{prior}\;\mathrm{odds}}{\underset{︸}{\frac{\mathrm{Pr}(H_{p}|I)}{\mathrm{Pr}(H_{d}|I)}}}\times \underset{\mathrm{likelihood}\;\mathrm{ratio}}{\underset{︸}{\frac{\mathrm{Pr}(E|H_{p},I)}{\mathrm{Pr}(E|H_{d},I)}}}=\underset{\mathrm{posterior}\;\mathrm{odds}}{\underset{︸}{\frac{\mathrm{Pr}(H_{p}|E,I)}{\mathrm{Pr}(H_{d}|E,I)}}}$$

where “Pr” denotes probability, “*H*_p_” the proposition summarising the prosecution’s point of view, and “*H*_d_” the proposition summarising the defence’s point of view. The letter “*I*” stands for the information mentioned in the section on the first principle of interpretation, and “*E*” (for evidence) represents the scientific observations (i.e., results or findings). The vertical bar “|” is called the conditioning bar and can be read as “given” or “assuming that”.

One of the most important lessons that can be learned from Bayes’ rule, as depicted in this form, is the nature of the roles played by different actors in the judicial process. It can be seen there are three terms in the equation, and the important question is: who takes responsibility for each of these three?

The first term represents prior odds, where the probability of each proposition given the information is considered. Assessing the allegations or the facts in issue is, without doubt, the duty of the fact finders. The last term also represents odds, which again relate to the probability of the propositions, but this time considering in addition the DNA results (or other forensic observations). These are said to be posterior odds, as they represent one’s updated belief after knowing the results (or the evidence) “*E*”.

The second term is the likelihood ratio (LR for short), which is a measure of the value of the findings. It is defined in terms of the ratio of two conditional probabilities: (i) the probability of the findings given that one proposition is true and given the conditioning information; and (ii) the probability of the findings given that the other proposition is true and given the conditioning information. The two conditional probabilities forming the LR may be assigned either on the basis of (published) data and/or the general knowledge (base) of the forensic practitioner. It is a measure of the relative strength of support that particular findings give to one proposition against a stated alternative [3,4,7,26,27]. A LR is a ratio of probabilities; thus, by definition, it is a number (as probabilities are numbers between 0 and 1). If the LR is 1, results are uninformative, and they do not support one proposition over the other. If the LR is larger than one, results support the first proposition compared to the alternative. If the LR is smaller than 1 (e.g., 0.001), then results support the alternative proposition over the first proposition. (It is sometimes reported that LRs can be negative, that is smaller than 0. This is incorrect. LRs theoretically can range between 0 and infinity. Log(LR) can be negative, but not LRs).

The focus of the LR is always on the findings, never on the proposition. It should be seen as a reinforcing or weakening factor in the perception of the truth of propositions that existed without the technical findings. This factor measures the change produced by the findings on the odds of the fact in issue being true. (In the literature, this factor is also more generally called a Bayes Factor (BF). With simple propositions, a BF reduces to a LR, as discussed in [28]. However, when multiple propositions are used, the BF does not reduce to the LR: it is a ratio of weighted likelihoods). Because the focus of the LR is on the scientific result, it is clearly in the domain and remit of the scientists. It allows them to provide assistance through the use of their expertise to assign probabilities for their observations given the truth of the competing propositions.

In addition, without considering Bayes’ rule, it appears quite sensible to say that scientists must give their opinion on the results and not on the disputed facts in issue. It is for the factfinder to render opinions on facts, and for DNA scientists to give the value of their results. When DNA scientists give their opinion on the alleged facts (or propositions) based only on the value of their results (i.e., LR), then they are said to transpose the conditional [26,27]. This is a very common error of logic and appears in many forms [29]. It has been called the “prosecutor’s fallacy” [30], but it is just as frequently to be found on the lips of defenders, judges, and journalists. It is more properly known as the “fallacy of the transposed conditional” because it is a matter of confusion between two conditional probabilities: the probability of the findings given the propositions and the probability of the propositions given the findings. One can erroneously transpose the conditional when giving an opinion on a single probability or on a ratio of probabilities.

## 5. Avoiding the Transposed Conditional

Not transposing the conditional is difficult, and it would be satisfying if one could avoid it just by knowing about Bayes’ rule and reporting a LR. It is so natural for the human mind to want an opinion on (alleged) facts, that it takes time and training to avoid this fallacy. There are several strategies that one can adopt to avoid this error of logic. Some of these were developed at the time when the Case Assessment Interpretation team from the former Forensic Science Service of England and Wales was training all their reporting officers; these are outlined in [6] and summarised below.

The first thing to investigate is whether the opinion pertains to the DNA results or to the proposition. The second aspect to check is that the sentence contains a word such as “if” or “given” and that these are associated with the propositions. If one has pen and paper, it is always a good idea to use notation, as spotting an error is then easier. Another coping strategy is to memorise correct and incorrect statements. When in doubt, one should re-phrase the statement of opinion along the lines of the correct format. Another strategy to ensure that there is no transposed conditional is to begin one’s sentence with the term “The DNA results” and add the probabilistic statement and the conditioning.

A lot of practice is needed to avoid inadvertently transposing the conditional: we discuss examples of incorrect formulations in Table 2. One should remember to state both propositions, as a LR is relative.

All incorrect/ambiguous statements originate from statements, judgements [31], or scientific communications. The last statement “The most favoured proposition is that S is the source of the DNA” is ambiguous: it could lead the reader to think that the proposition which is the most favoured is the most probable. This is not the case, as depending on prior odds, the most probable proposition might not be the one that is the most supported by the results. An example is shown in Table 3 using Bayes’ rule. One can see that with a LR of 1 million and very low prior odds of 1 to 10 million, the posterior probability of the first proposition would be 9% (or 0.09); the most favoured or most likely proposition would be the alternative.

One can note that, if the prior probability of the first proposition is zero, then whatever the LR, the posterior probability is zero as well. It would be rare to have this situation. According to [14], when assigning our probabilities, we should admit the possibility that we might be wrong. If we do, this rule denies probabilities of 1 or 0. (This rule is called Cromwell’s rule, named after Oliver Cromwell, who said to the Church of Scotland, “I beseech you, in the bowels of Christ, think it possible you may be mistaken”. Calling it Cromwell’s rule is attributed to Dennis Lindley). The probability of an event given K is 1 if, and only if, K logically implies the truth of E (and of zero if K implies the falsity of E). To provide an illustration where there can be a prior probability of zero, we revisit the case where the issue was whether the urine was from the athlete or not. You will remember that the DNA was different from the athlete’s DNA, but that the donor and the athlete shared many alleles. In that case, it was reported that the DNA profile comparison was of the order of a billion times more likely if the person’s sister was the source of the urine, rather than if an unrelated person was. The DNA scientist reported that the probability that the urine originated from the sister was 99.9999999%. In those conditions, they conclude that it was practically proven that the urine was from the sister. This prompted the athlete’s lawyer to write a letter to say that there was only a minor problem in that reasoning: the athlete did not have a sister (but she did have a half-sister). In this case, the probability of the proposition is zero. This also shows why it is best for scientists to give an opinion on the results, however large their LR. It is generally not considered the scientists’ remit to give an opinion on facts (except, as mentioned earlier, in some countries for paternity cases), which is the case if they give posterior probabilities.

To calculate posterior probabilities using Bayes’ rule in odds form (Equation (1)), one multiplies the prior odds by the LR. This gives us posterior odds. If the odds are a to b, to obtain the probability of the first proposition, one divides a by (a + b). If the odds are, for example, 1:1, the probability of the proposition is 1 divided by 2, thus 0.50 (or 50%, as probabilities can also be expressed as percentages).

It should be noted that, when being extra careful about not transposing the conditional, some think that it is incorrect to say “the results support the first proposition rather than the second”. The use of the word “support” in this context was proposed in a manner analogous to H. Jeffreys [32]. It does not indicate that one proposition is more likely than the other, only that the results are more probable if the first proposition is true than if the alternative is. Because it is important to indicate what our results mean and do not mean, we recommend outlining this point in our statements.

## 6. Hierarchy of Propositions

The concept of a hierarchy of propositions [33] applies to all forensic disciplines. It was developed initially for evaluative rather than investigative opinions, and one will note that the examples of propositions given in the early Case Assessment and Interpretation (CAI) publications were generally suspect-focused. Over time, the CAI development team found that the concept of a hierarchy of issues and propositions was equally applicable to investigative issues [16]. The classification of propositions into three main levels (source, activity, and offence) allows forensic scientists to contextualise the results, to consider the factors in their evaluation and to communicate to the client the purpose of the proposed examinations. The demarcation between the levels is not meant to be rigid, and it is recognised that sometimes levels will be difficult to distinguish. The levels simply provide a model framework that helps scientists to organise their thinking, actions, and decisions. The important point to stress to practitioners is: do not try to force all the issues you will encounter into one of the levels of the framework. Instead, just specify clearly in words the issue, and hence the propositions to consider. If the issue and propositions then fall neatly into one of the categories (i.e., one of the levels), so much the better, but do not worry if the issue/propositions do not seem to fit one of the categories. The important thing is that you have clearly specified the issue, with which the examinations can help, both for yourself and for the factfinders.

The hierarchy of propositions is generally presented from source to offence (see examples in Table 4). It is structured in a hierarchy as scientists will need more case information and more knowledge to assess their results given offence- or activity-level propositions, compared to those of the source level. It is important to outline that when identifying the level in the hierarchy where they can be the most helpful, experts shall not stray outside the bounds of their expertise, and value must be added. This is done by bringing knowledge that is needed for understanding the meaning of the results in the context of the case and that otherwise would remain unavailable to the court.

### 6.1. Issue with Which the Forensic Scientist Can Help: Is Mr Smith’s the Source of the DNA/Biological Material?

When the hierarchy of propositions was first suggested, it was only possible to obtain a DNA profile from biological fluids present in relatively large quantities. In such cases, one could reasonably assume that the DNA profile was derived from a known biological fluid (e.g., blood). This assumption became questionable with the advent of more sensitive techniques. This led to new levels (still contributing to answering the question of the source): the DNA level (or sub-source level) and the DNA contributor level (or sub-sub-source level [34]), for situations where the issue is whether a person is the source of part of the DNA mixture (e.g., a major component). We discuss below when it is meaningful to choose source-level propositions or their associated sub-levels.

#### 6.1.1. Source-Level Propositions

Source-level propositions are adequate given two conditions: first, the issue should be whether a given person is the source of the material. Second, there should be no risk for the court to misinterpret the findings in the context of the alleged activities. This would typically be the case when the material is found in such a quantity that there is (i) no need to consider its presence for reasons other than the alleged activity (i.e., it will be accepted by the fact finder that the material is relevant), and (ii) that the nature of the material can be safely assumed. The following example is adapted from [9], illustrating when considering the results of a DNA comparison given source-level propositions is not misleading:

“A large pool of fresh red bloodlike material is recovered at the point of entry at a burglary scene and the circumstances suggest it has originated from the offender(s), whomever they may be. A sample is delivered to the laboratory for DNA analysis. Combination of a positive presumptive test, large quantity and appearance allows the scientist to safely assume that it would be agreed that the stain is blood. The defendant, Mr D., says that he has never been in the premises and denies that the blood at the scene is from him. The set of propositions can be (1) the bloodstain came from the defendant and (2) the bloodstain came from an unknown individual.” Assuming that the nature of the material will not be contested, the same term, “bloodstain”, can be used in both propositions. It is sometimes believed that source-level propositions can be formulated when the nature of the material (e.g., blood, semen, saliva, cellular material) is disputed and that this accommodates consideration of the probability of presumptive tests. However, it does not as the probability of observing a positive (or a negative) result for the presumptive test would be equivalent whether the blood is from Mr D or from someone else. Therefore, if the nature of the material is disputed, it is, in general, more meaningful to consider activity-level propositions [11]: these will take into account the results of presumptive tests as well as the transfer, persistence, and the presence of the material as background (i.e., material from an unknown source present for unknown reasons).

#### 6.1.2. Sub-Source-Level Propositions

The advent of highly sensitive methods has made it possible to produce DNA profiles from very small quantities of biological material [35]. With invisible or small stains, the nature of the material from which the DNA profile is produced is often unknown. In such cases, if the issue is who is the source of the DNA, one can assess the results of a DNA comparison given sub-source propositions (i.e., the source of the DNA, not of a given body fluid). These are especially useful for producing investigative leads. As mentioned in [10], one can use a LR in both the investigative and the evaluative phase. The main difference is that in the evaluation phase, there will generally be a suspect/defendant around whom the issues and propositions will be defined. In this situation, it will be necessary to take into account an alternative proposition, typically the defence’s view of events, if that has been communicated to the scientist. This person may, for example, mention that they know the victim. Again, in such cases, assessing the results given activity-level propositions will generally be more meaningful. However, the DNA scientist operates in “investigative mode” where, for example, a database search is carried out because there is no suspect for the crime. Here, in the initial phase, what is of interest is to provide information about who could be the source of the DNA. An example of sub-source propositions would be: “The DNA is from candidate X” or “The DNA is from an unknown person”. If the aim is to produce a useful lead, the person will not have been arrested yet, and de facto, the scientist will have been provided with no alternative proposition or information. The person making the investigative decision (here, for example, to arrest the candidate or not) will not be the court but, in some jurisdictions, an investigating magistrate, or in others, a police investigator. When there is very little case information, the value of the comparison needs to be much higher for the person making the decision (e.g., to arrest the candidate or not). In addition, for cost effectiveness, one may also want to avoid investigating many false leads. This explains why larger LRs will often be needed for investigative purposes.

#### 6.1.3. Sub-Sub-Source-Level Propositions

If the issue is whether a POI is the major (or minor) contributor of a DNA mixture, then one can consider sub-sub-source propositions [34]. An example of sub-sub-source propositions would be: “Mr A is the major contributor to the DNA mixture” or “An unknown person is the major contributor to the DNA mixture”. If it is important that the POI is compatible with the major component, then this generally is an indication that the issue lies in the activities and that scientists can add value by considering activity-level propositions. If the relative quantity is not an important factor, then sub-source propositions are generally preferred to sub-sub-source, as the former allow accounting for all the results (and not only part of the mixture).

### 6.2. Issue with Which the Forensic Scientist Can Help: Did Mr Smith Perform the Activities Alleged by the Prosecution or Those Alleged by the Defence?

The next level in the hierarchy of propositions is the activity level. The evaluation of given activity-level propositions generally involves assessment of the extrinsic characteristics (e.g., quality of the DNA profile, relative quantity of DNA, where the DNA was sampled from) and should be considered when transfer, persistence, or background have a significant impact on the understanding of the value of the findings in the context of relevant case circumstances and the alleged activities [9,11,36]. Depending on the case and the information content of the profile, the source of the DNA might be contested or agreed. When the source of the DNA is not contested, but the activities leading to the deposition of the DNA are, one does not necessarily need to consider the source of the DNA anymore For investigative purposes, the DNA profile of the trace and of the person still need to be compared. To determine the value of this comparison, one will assign a LR given sub-source-level propositions. However, if there is no dispute and thus only one proposition (i.e., the DNA is from the POI), this LR value is not relevant. It is the LR given activity-level propositions that is meaningful. It is sometimes believed that to consider activity-level propositions, one needs to agree on the source of the DNA. This is not true: in this type of evaluation, one can consider both possibilities (i.e., that the DNA is from the POI or not). If so, one has associated sub-source propositions. However, depending on the rarity of the DNA profile, this consideration will have little impact on the value of the findings given activity-level propositions. Indeed, as indicated by the England and Wales Court of Appeal [37]: “It makes the task of the jury so much easier if they do not have to plough through and listen to evidence that is simply not in dispute.” Let us look at an example where the issue is one of activity. Assume a stolen car crashed into a group of pedestrians, killing one and injuring others. Two people escaped from inside the car and ran away. Acting on information, the police quickly arrested two men, Mr Smith and Mr Jones. Both admitted to being in the car at the time of the collision, but both denied being the driver, instead accusing the other of being the driver. The issue would be: “Did Mr Smith or Mr Jones drive the car at the relevant time?”. The activity-level propositions would be: “Mr Smith drove the car at the relevant time and Mr Jones was the passenger.” The alternative would be the reverse: “Mr Jones was the driver and Mr Smith the passenger.”. As both the driver’s and the passenger’s airbags were activated at the time of the collision, an examination of the airbags for biological material could help to address the issue. Another typical case where sub-source-level propositions might not be meaningful, would be when a person has legitimate access to the object or person on which examinations have been performed (e.g., a gun found in the POI’s car). In such cases, the source of the DNA (i.e., the person) may well not be contested (which does not mean that the DNA does not need to be analysed, only that, depending on the results, these may not need to be assessed). It is worth noting that activity-level propositions allow for the assessment of the absence of evidence [33,38]. The saying “The absence of (matching) evidence is not evidence of absence” is not always true. To know when it is, formal evaluation is needed. (As much as possible we try and avoid the term match for two reasons: first, laypersons believe that saying there is a match means that the DNA is from the person; second, scientists can at best state they were unable to see any difference they judge relevant. This does not mean that the two profiles are identical: indeed, two separate identities cannot be identical because, given sufficient resolution, all distinct entities are distinguishable from each other, even when two items come from the same source). Activity-level propositions also facilitate the combination of DNA results from different items that were touched because of the same activity (e.g., the two airbags, and possibly the steering wheel, in the example above). Finally, one should note that sometimes it may be difficult to distinguish offence from activity. As an example, a proposition such as “Mr A stabbed Mr B.” or “Ms A shot Mr B.” may be considered either as an offence and/or an activity-level proposition. Remember, as indicated previously, the lines of demarcation in the hierarchy should not be seen as rigid: it is meant to organise thinking, actions, and decisions.

### 6.3. Issue with Which the Forensic Scientist Can Help: Is Mr Smith the Offender or Does He Have Nothing to Do with the Offence?

The issue for the court is always the offence, which is at the top level of the hierarchy. An example of a pair of competing offence-level propositions could be: “Mr Smith committed the burglary” and “Mr Smith had nothing to do with the burglary”. It should be remembered that propositions and case information are closely entwined so that in the case information, more detail would be given indicating, for example, that Mr Smith visited the jewellery store 3 days prior to the burglary. It is sometimes cautioned that offence-level propositions are not the domain of the scientist but of the court. Although this is true, this statement applies to all levels of propositions, as scientists offer their opinion on the results and not on propositions (or else, they would transpose the conditional). This is valid whatever the level, and offence level is not special in that sense. However, the evaluation of findings given offence-level propositions is special in the sense that it is rare that forensic scientists add value by considering propositions at the offence level, compared to a level lower in the hierarchy. Indeed, in many cases, the difference between activities and offence lies in the intent or consent, and in that case, obviously, biological results cannot help discriminating between these two levels. A typical case where DNA scientists cannot add value would be if they considered “rape” instead of “vaginal/penile penetration or consensual sex” [39]. Biological results do not give any information on the issue of consent, pre-meditation, nor intent, thus DNA scientists cannot help the court address those issues. In these situations, DNA scientists should not rise in the hierarchy, as they would not use any specialised knowledge, nor add value, when considering the offence rather than the activities.

However, the consideration of offence-level propositions allows adding value when there are multiple forensic findings that need to be combined by a forensic scientist. Such a case could be a burglary implying multiple activities: for example, breaking glass, jumping out of window, opening a safe. In this situation, offence-level propositions would enable combining the different results (e.g., shoe-marks, fibres, and DNA profile comparisons). The list of the activities would be very similar to what constitutes an offence in that case. Offence-level propositions have also been proposed to explore the impact of other factors such as relevance [40,41].

## 7. Formulation of Propositions

Within a forensic case, people (e.g., the police, the defence, or witnesses) will make various claims or statements. These are either true or false and can be affirmed or refuted. It is once they are formalised by the scientist that these claims will be referred to as propositions. (Some people use the term hypothesis to designate propositions used for the evaluation of findings. We prefer to keep this term for situations where scientific experiments are performed to “test” a hypothesis. As discussed in [3], this enables distinguishing between both concepts, and it is only when a proposition is formulated for empirical testing that we will call it a hypothesis). As described in [33], propositions need to be mutually exclusive (if one is true, the other is false) and formulated in pairs (e.g., views put forward by the parties to the cases) against a background of information and assumptions. They should also be amenable to a reasoned assignment of credibility by a judicial body [9]. There may be more than two propositions, but in the context of a criminal trial there will be two views. For the formalisation of propositions, the basic criterion is that they should be formulated in such a way that it is reasonable for the scientist to address a question of the form: "What is the probability of the observations given this proposition and given the framework of circumstances?” [12]. There are other important criteria to keep in mind when formulating propositions. For example, propositions are about causes and as such will be assessed by the decision maker (e.g., factfinder). If propositions contain factors that are to be considered in the evaluation, these factors cannot be assessed by the scientists anymore: being part of the proposition, they will be assessed by the decision maker. Formulation of propositions needs to adhere to specific criteria and appropriate phrasing. As it is a difficult task that requires expertise, the DNA scientist is in the best position to formalise the propositions, and one cannot expect that prosecution or defence formally define the so-called “Prosecution or Defence propositions” themselves. (The defence proposition may be compound. For example, if the alternative is that the DNA is from an unknown person, this unknown person may be unrelated or a sibling or a cousin). These terms are used to indicate that the propositions represent the views of these two parties as understood from the case information available (i.e., that the propositions were formulated against the background of information available from the parties). However, should one of these not accept the propositions considered by the scientist, a new evaluation will be needed. Ideally the formulation of propositions should be discussed between the parties and the DNA scientist before doing the work.

There have been many publications on the formulation of propositions [10,11,18,23,33,42,43,44,45,46]. In Table 5, we give the criteria to which they should adhere and examples of poorly worded propositions, and their corresponding, more meaningful formulations are discussed in Table 6. It is important to emphasise that case information and propositions are entwined and will both appear in a statement. What goes in the information and the proposition will depend on the case. However, because we repeat propositions in our statements and in court, it is preferable to keep them short and snappy.

In the evaluative stage, propositions should not be findings-led, and thus, ideally, the formulation of propositions should be made without knowing the results. This is an essential, early component of the recommended process of Case Assessment and Interpretation (mentioned later in Section 11 on Pre-assessment). In DNA casework—especially for investigative purposes—when considering sub-source propositions, the alternative source is generally by default an unknown person. If the propositions are standard and if knowing the findings does not impact the value of the comparison, then it is acceptable. However, if there is an impact, it is more problematic. An example, of a findings-led proposition could be “Mr A is the minor contributor of the DNA mixture”, because Mr A happens to be compatible with the minor contributor. Another could be to change the number of contributors because there is one exclusion at a locus if the mixture is considered as a two-person mixture, but not if it is assigned as a three-person mixture. Regarding the number of contributors to a mixture, one should note that there is no need to consider a specific number. It is sensible in some cases to consider a variable number of contributors [44,47]. A last example of findings-led propositions would be the situation where there are two candidates for the same mixture. If one chooses a different set of propositions based on whether they explain the mixture together or not, then the propositions could be findings-led. In such cases, a suggested solution is again to consider multiple propositions [48].

## 8. Formulation of the Alternative in the Absence of Information from the Person(s) of Interest

A POI is under no obligation to provide information and may give a no-comment interview. In such a case, scientists will formulate an alternative that appears the most reasonable based on what they know [9,12]. The investigation might provide information, for example that the victim and defendant visit the same gym, suggesting that activity-level propositions might be appropriate. One can also suggest the negation of the first proposition (e.g., Mr Smith is not the source of the DNA), provided we are explicit on what is meant by “not” [46]. This ought to be explained in the paragraph describing the case information. The implication of adopting such a negation should be set out clearly for the receivers and users of the opinion—it tends to maximise the value of the observations in support of the main proposition over the alternative. Some scientists do point this out in their reports. The important considerations regarding, for example, from whom the DNA comes (e.g., if not from Mr Smith) will be clearly disclosed. A caveat should indicate that should these assumptions not be relevant to the case, a new interpretation and perhaps further analysis will be necessary based on the new case information and new alternative.

## 9. Distinction between Explanations and Propositions

It is particularly important to distinguish propositions from explanations when the issue is activity, as these explanations are more and more commonly offered by the parties. In the context of the Case Assessment and Interpretation model [2,18], explanations have been recognised as intermediate considerations when exploring less formal alternatives. Explanations can be very useful in the investigative stage: they provide new leads and outline what information is needed. Explanations will be generated based on the observations and generally do not qualify as formal propositions for the evaluative stage. If the explanation is prescriptive, the probability of the observations given this explanation will be one. Examples of prescriptive explanations could be “The trace has been contaminated with the suspect’s DNA”, “The persons were in contact recently and transferred DNA directly,” or if we want to state the obvious, “The stain came from someone with the same DNA profile”. Explanations may be speculative or fanciful. Contrary to propositions, they do not depend on case information and are not necessarily mutually exclusive. Examples of various types of explanations are given in Table 7.

In the context of DNA, especially when the issue is how the DNA was deposited, one should avoid, or at least outline the limitations of, considering explanations in court: in this process the scientist cannot assign the value of the results which then could easily be misunderstood. As indicated in [49], “‘bare’ explanations are likely to be of limited assistance to fact-finders, and might even be regarded as potentially misleading and, sometimes, pernicious.” We will revisit this aspect in the section on communication and reporting.

## 10. A Note on Multiple Propositions

While it is not feasible to achieve absolute exhaustiveness, it is important that all the relevant case information is considered when formulating the propositions. This can imply having two main propositions, but several sub-propositions. Indeed, if the scientist omits a proposition that is relevant, it is possible to have results that support a proposition that would not be supported if all pertinent information had been considered. In the context of mixtures, one may have to consider different numbers of contributors [44,47], different persons of interest [48], or different degrees of relatedness between the POI and the alternative source and/or different populations [50,51]. It has also been shown that for close relatives, considering multiple propositions (but two views) achieves better sensitivity and specificity [52]. An example of multiple propositions (two main propositions and associate sub-propositions) with two persons of interest—Mr A and Mr B—could be for the evaluation of the comparison of Mr A’s DNA profile with a two-person DNA mixture:

- Mr A is a contributor
  - Mr A and Mr B are the source of the DNA mixture.
  - Mr A and an unknown are the source of the DNA mixture.
- Mr A is NOT a contributor
  - Mr B and an unknown are the source of the DNA mixture.
  - Two unknown persons are the source of the DNA mixture.

## 11. Pre-Assessment

With the segmentation of forensic science and the use of DNA databases for investigation, it is not always realised how crucial case information is for devising an appropriate case strategy and for giving meaningful answers. One important outcome of the Case Assessment and Interpretation project was to formalise what is known as case pre-assessment [49,53,54]. Most of the stages described imply thinking and communicating about the problem before proceeding to examinations and the commitment of resources; one can argue that it just reflects good forensic practice.

### 11.1. Revisiting Good Forensic Practices for Evaluative Reporting

To deliver the best service, the first stage will be to define the needs in the case and explore how one can help with the issue. Once the questions have been clearly identified and discussed with the mandating authority, an effective examination strategy can be devised and agreed to with the client, the work can be performed, and the results and subsequent interpretation reported. This process appears straightforward, but it can be difficult to apply in practice. First, because the police will not always be taught how important case information is to help define the best examination strategy, they might not be aware of some limitations. Moreover, in many cases, the work will first be performed to provide an investigative lead. Once the lead has been produced, the criminal justice system might not always be aware that the case information provided by the POI can drastically change the value of the results.

Efficient case management cannot proceed without task-pertinent information, such as the allegations that are contested and those that are not, what the persons of interest say (if available), or where and how the items were recovered (e.g., inside the car of the POI). The case circumstances help the scientist to understand the issues and identify what types of opinion (investigative or evaluative) they should offer. Administrative information, such as the deadline and the budget, will inform the choice of the examination strategy. Thinking of the case and of one’s expectations before doing the actual work has many advantages, including ensuring (i) that the work done is meaningful and cost-effective, (ii) that the scientist thinks and writes down the expected results given the truth of each proposition, helping to mitigate post-hoc rationalisation (or bias). Indeed, even the most logical of scientists, once confronted with the results, will tend to rationalise their expectations; these will appear more likely once they are observed. Having to think of the range of different outcomes also ensures that the probabilities assigned are coherent. Pre-assessment is particularly valuable when DNA scientists need to consider phenomena such as transfer and persistence.

### 11.2. An Example of Post-Hoc Rationalisation That Can Be Avoided Using Pre-Assessment

Let us imagine that we have a case where it is alleged by prosecution that Mr S digitally penetrated Ms J’s vagina. Mr S says that he only spent the night taking care of Ms J as she had drunk too much alcohol. DNA swabs are taken from Mr S’s nails 3 h after the events. Assume we did not carry out any case pre-assessment (i.e., we did not set out, broadly, all the potential outcomes of the examination and, more importantly, their probabilities). Assume that we now know that a full female DNA profile was produced from the swab from the right hand, we could be tempted to say that this was an expected outcome (i.e., there was a high probability that we would have obtained this outcome if the prosecution proposition were true) or “within the range of our expectations”. However, if a partial female profile had been obtained, we could be tempted to say that one needs to consider the possibility that Mr S has washed his hands. If that were true, then we would have expected a partial profile. So, whatever the outcome, there is a temptation to rationalise it. Had we not known the outcome, then we could not be biased by it. The same can be said for the POI—once confronted with the results, they might be tempted to rationalise the findings. For this reason, one should ideally: (i) assign the probability of the possible outcomes without any knowledge of the actual results (this can be done by another scientist unaware of the findings, or using a previously built Bayesian Network [55]), (ii) ask the parties their version of events, without mentioning the results. This should theoretically not be problematic, as the alleged facts should not depend on the results.

## 12. Communication, Reporting, and Testimony

To exchange information, the messenger (e.g., the DNA scientist) needs to convey the value of the findings clearly, and the recipient of information (e.g., judiciary, police investigator) needs to understand the message in the intended manner. This implies that DNA scientists explain their reasoning and the meaning of the results in an accessible way to a large audience, who will have different education, backgrounds, languages, and expectations. As discussed in [56], more research is needed to address the effective presentation of forensic findings. In this section, we briefly explore the topic, but are of the opinion that, to tackle this challenge, DNA scientists should master the key concepts of interpretation. This will be easier if they have received formal education in forensic interpretation.

For efficient communication, it is also important to adapt to the audience. As alluded to earlier, the recipient of forensic information does not come without preconceived ideas: in one case, they may have the impression that DNA is unique; science is exact, precise, and gives answers that are independent of human judgement. In another case, they may think that DNA is too complex and cannot be helpful. There can easily be a disconnection between prior (mis) knowledge and the reality of forensic DNA interpretation. Another point is that, if communication takes place in the context of a given case, then according to [57], “the audience’s pre-existing beliefs or attitudes towards the communicator, topic or object of uncertainty might influence or change the effects of uncertainty communication”. Thus, not only will the communicator have a bearing on how the value of the evidence is perceived, but so will the other case information available to the recipient of information. For these reasons, formal education outside the courtroom is certainly the best means to ensure that the nature of forensic opinions is well communicated and understood. Having forensic interpretation courses in the curriculum of law degrees, as is the case in some universities (e.g., Lausanne, Switzerland), helps ensure that the future judiciary and advocates understand key concepts such as uncertainty, probability, and likelihood ratios.

The provision of a common language contributes to the improvement of communication: explaining what we mean (with the use of a glossary) as well as what we do not mean is key. Moreover, one needs to acknowledge that some words (e.g., guess, subjective, match, assumption) come with a strong connotation and are prone to misunderstandings: as such, they should be avoided whenever possible or be explained.

### 12.1. Reporting: General Desiderata

**Figure 1:**
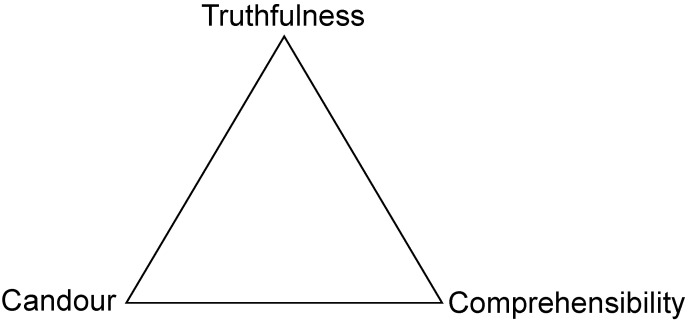
Triangle representing the tensions between the goals of communication.

The desiderata, as well as the principles of interpretation, that have been proposed for evaluation of the results apply to reporting (orally or in writing). In addition, it has been proposed that the goals of communication should be truthfulness, candour, and comprehensibility [23]. The pursuit of one goal often requires the sacrifice of the others. The tension between these three goals can be illustrated by a triangle where each goal represents an angle (see Figure 1, Buckleton, personal communication). Candour implies that experts adhere to a code of conduct and only report in their area of expertise.

### 12.2. Statement Writing

The exact requirements for statement writing will depend on the jurisdiction, but guidelines for good practice [9,21] should be followed. These provide suggestions regarding the content of the statement and caution the reader against certain words and phrases still in use today (e.g., consistent with, association, link, contact, support or refute. Because refute is a categorical statement, it is not considered as the converse of support. There are degrees of support, but not of refutation. Instead of writing “whether the results support or refute”, one could say “whether the results support one proposition compared to the alternative or neither proposition”.) Because of their nebulous meaning, these terms generally poorly convey the value of the findings.

Because the value of the results depends on the scientific knowledge of the DNA scientist and the task-pertinent case circumstances, statements should include a specific paragraph describing the information that was provided, as well as the assumptions made. The issue should be clearly identified, propositions formulated, and scientists should state their willingness to help address other alternative propositions if the framework of circumstances changes or if it has been misunderstood. As the case information is not a collection of “facts”, it is important to indicate that should the case information change, it will be necessary to review the interpretation. Following the description of the items, methodological aspects sometimes referred to as “Technical issues” (e.g., methods of analysis and interpretation, aspects regarding transfer, persistence, and recovery of DNA) will be described. The results of the analysis will be presented and evaluated. In complex situations (e.g., when assigning the value of the findings given activity-level propositions) a paragraph pertaining to the probability of the results given the case information and given each proposition will allow the reader to understand the basis of the reasoning and the source of the data. Reporting what a LR is and what it is not should help convey the difference between the probability of the findings (given the propositions and information) and the probability of the propositions (given the findings and the information). The limitations of the interpretation and the issues considered should also be clearly stated. In Table 8, we suggest examples of useful caveats.

### 12.3. The Use of Verbal Equivalents

To communicate the meaning of the LR, verbal equivalents (or verbal scales) have been suggested [4,58,59]. If a quantitative LR is presented, the LR value shall come first [9] and can be accompanied by a verbal equivalent [56,59,60]. An example could be: “A likelihood ratio of the order of 1000 has been assigned, which means that the DNA results are 1000 times more probable if Mr Smith is the source of the DNA rather than if his unavailable brother is the source. Therefore, the DNA findings support the proposition that Mr Smith is the source of the DNA rather than his unavailable brother. The level of support can be qualified as strong.”. If one understands what a LR is, verbal equivalents are superfluous. To show how beliefs should be theoretically updated based on the DNA results, it might be more efficient to include a table with a few examples of prior odds and the effect of the results (i.e., the LR obtained) on posterior odds. (For persons who are unfamiliar with odds, it is also possible to transform odds into probabilities.) This also has the advantage of showing the difference between LRs, prior odds, and posterior odds.

It is sometimes believed that one needs different verbal scales for DNA (more precisely, for autosomal DNA) because LRs are much larger than those of say mtDNA or another discipline such as fibres. This is not the case: an explanation of why some DNA scientists believe that a DNA comparison with a LR of 1000 is uninformative is that DNA scientists nowadays work mainly in the investigation phase. In such a situation, larger LRs are generally needed to avoid having too many candidates from the national DNA database (and too many false leads). In addition, if there is little additional case information, then the authority may need more discriminative results to decide if a person should be arrested or not. Another (non-mutually exclusive explanation) could be that DNA scientists are so used to having very large LRs that they are like billionaires: 1000 could thus be perceived as “pocket money”. As outlined by several authors [4,9,61], one should adopt the same verbal equivalents whatever the discipline and whatever the results (autosomal DNA, Y-STR, or mtDNA comparisons). It follows that one needs the same verbal scale whether reporting results given activity- or sub-source-level propositions and whether the results are to be used for missing persons, paternity, or sibling cases. There has been much discussion on verbal scales [21,58,59,60,62,63]. There are three points where there seems to be a consensus: (1) If the LR value is “one”, then results must be considered as uninformative (it is the results and not the LR that is uninformative. Similarly, it is the results and not the LR that provide support for one proposition compared to the other. The LR indicates the magnitude of the support given by the results to one or the other of the propositions). Therefore, the observations provide no assistance in addressing the stated propositions. (2) If LRs are larger than one, the results support the first proposition. (3) When LRs are smaller than one, the results support the alternative. Because verbal scales are based on a convention, it is difficult to achieve standardisation: different scales have been proposed with different resolutions (number of intervals) and terminology. Although verbal equivalents are unsatisfactory, concentrating on the LR value alone does not look very promising either, and it has been shown that people have difficulties understanding the concept of LRs in isolation. While no one has yet identified the ideal method for the presentation of the value of scientific findings, this does not mean we should change the method of evaluation (we would not change the methods of analysis because people do not understand DNA analysis). However, until there is more widespread understanding of the concepts of LR and probability, if the DNA scientist thinks it is desirable to add a verbal equivalent, this is not harmful provided that the LR value is given first.

### 12.4. Testimony

Questions in court relate more and more as to how the DNA was deposited. However, few DNA experts will prepare a report on this topic and some are tempted to wait and answer questions in court. This is not ideal [12,64], because evaluation of biological results is difficult. As such, it should be submitted to the same criteria as any other evaluation (i.e., be the subject of a written statement that has been peer reviewed and is available to the defence).

In court, DNA scientists may be asked to report the value of the results given sub-source propositions, and then answer hypothetical questions, which generally are explanations rather than propositions. An example could be: “What if the defendant had shaken hands with the real offender, would it not be possible to recover in such a case DNA from my client?”. One might be tempted to say, yes, this is possible. However, as stated in [65] “if at the end of the expert’s evidence, the fact-finder is left with, on the one hand, an impressive big number (the LR given sub-source propositions) and on the other hand, a list of possible explanations for the transfer (as a result of specific activities), how do they decide what the DNA evidence means, and how does the evidence impact their decision?”.

Questions about the possibilities of DNA transfer require a more subtle answer. In general, when a case comes to court, DNA scientists will help the triers of fact in a more meaningful way if they assess the value of the DNA results in the context of the case. One must also be aware that if answering questions such as “Is it possible that there was secondary transfer?”, one would be giving an opinion on the alleged activities implying secondary transfer. Similarly, if one comments on the origin of the DNA and says that “The most likely source of the DNA is the vagina” [37], then if one does not specify how prior odds were assigned, it is unclear if it is a transposed conditional or a posterior probability. If the nature of the fluid implies an activity (as would be the case for vaginal cells), this is problematic, as the nature of the material relates directly to the issue that is of the remit of the court.

If the questions regarding how DNA was transferred appear reasonable to the court, it is recommended to ask for a recess [12,25] and perform a proper evaluation of the results given what is alleged.

If DNA scientists cannot assess the value of the observations, for example, in light of the proposed activities, because there is no time, a lack of case information, or a lack of knowledge, then, provided the person is qualified, they should outline that without further information, the findings should be considered uninterpretable (thus uninformative) and presumably inadmissible, because of the risk of their being prejudicial.

## 13. A Discussion on Likelihood Ratios

Now that you are familiar with the main concepts of interpretation, we would like to discuss aspects relating to what LRs are, if they can be estimated, how precise they should be, and whether it is problematic to transpose the conditional when LRs are very large.

### 13.1. Likelihood Ratio and Probability

Scientists should know how to explain what a likelihood ratio is. As described earlier, a LR is a ratio of two conditional probabilities given competing propositions and conditioning information. However, what is a probability? A probability is a number between zero and one that represents our uncertainty with regard to the truth of some aspect of the universe in the past, present, or future. All probabilities are conditional: they depend on what you know, what you are told, and what you assume. In some simple situations, such as coin-tossing, there might be little disagreement between people about the truth of a statement such as “the coin will land showing a head”. In general, though, the central component of knowledge means that different people will have different probabilities for the same thing. We say that probability is *personal*. In relation to any uncertain event there is no “correct” probability, so we say that probabilities are “assigned” rather than “estimated”.

Because a likelihood ratio is the ratio of two probabilities, it necessarily follows that a LR is personal also. Thus, in relation to any particular pair of propositions in a DNA case, there is no “correct” LR: different scientists will bring different levels of knowledge (and understanding) to bear. What we require is that the scientist to whom we pay attention is *well calibrated*: that is, we expect that the scientist can be relied upon to assign large values to the LR in cases where the prosecution proposition is actually true and to assign small values to the LR in cases where the defence proposition is actually true. This concept of calibration is critical to software systems that are created to assist scientists in assigning LRs and to the publications supporting advanced DNA probabilistic genotyping calculations, include extensive studies of *ground truth* cases. A ground truth case is one that has been artificially created; in its simplest form, it will consist of two sets of data and the truth with regard to whether the two are from the same original source (*H*_p_ true) or not (*H*_d_ true) is known without doubt. The distribution of LRs from *H*_p_-true ground truth cases should peak at high values (greater than one); the distribution from *H*_d_-true ground truth cases should peak at small values (less than one). If considering, for example, the source of a DNA mixture recovered on a scene, because people can share part of their DNA, it is expected that some non-donors will share alleles adventitiously and have a LR larger than one fortuitously. This is even more true when comparing mixed/partial DNA profile with individuals who are closely related to contributors or due to co-ancestry effects [52]. There are sophisticated measures for assessing calibration experiments quantitatively, but one simple one is known as “Turing’s rule”. This follows from a theorem, attributed by IJ Good to Alan Turing [66,67], that shows that the “expected value of a LR in favour of a wrong proposition is one”. This implies that the average value of the LR in a large *H*_d_-true calibration experiment should be approximately one: in practice, this means a highly skewed distribution, with most LRs close to zero and the odd one or two substantially greater than one. For example, suppose that a LR of a million is computed given sub-source propositions. It follows that if we simulated profiles of millions of non-donors, we would expect roughly 1 in every million non-donor DNA profiles to yield a LR of 1 million when compared to the DNA profile of the trace. We would also expect 999,999 out of every million non-donor profiles to yield a LR of 0 when compared to the DNA profile of the trace. The average LR of these ground truth comparisons is then 1.

Good (ibid) also showed how he and Turing regarded the logarithm of the LR as a measure of evidential weight, and this view appears highly relevant to the forensic field. This notion contributes to the idea of expressing verbal conventions in rough correspondence to orders of magnitude of the LR [58]. It also suggests the notion that precise values of the LR are not needed for effective communication of evidential weight to jurors. Hence, our suggestion is to report LRs to one significant figure. If a LR of 10,256.32 were computed, we would conclude that the findings are of the order of 10,000 more probable if… than if…This allows us to convey that it is the order of magnitude that is important.

For LRs smaller than one (i.e., when results support the alternative), it has been suggested to reverse the propositions and adopt the same rounding rules. Indeed, numbers smaller than one are difficult to understand: for example, if our LR is 0.01, shall we say results are 0.01 times less (or more?) likely if the first proposition were true rather than if the alternative were true? It is easier to report that results are 100 times more likely if the alternative proposition were true rather than if the first proposition were true.

Current population genetic models condone the multiplying together of genotype probabilities across many loci, and this in turn leads to extremely large LRs—billions, trillions, quadrillions, and still further. When this issue first arose in the UK, Foreman and Evett [68] argued that the extent of investigations into the independence assumptions implicit in this process was insufficient to justify the robustness of LRs in excess of one billion. So, when the Forensic Science Service introduced the ten locus STR system into casework, the largest magnitude of LR quoted was one billion. This was expected to be a temporary expedient, but to this day, there still seems to be little in the way of large-scale between-locus dependence effects. This seems to cause little embarrassment, and it is common to see LRs of the order 10^20^ reported in DNA cases (It should be noted that with very small numbers, there are some analogies that are misleading. An example is the “several planets earth” or the “galaxy” argument used, for example, in the Jama Case [69]. “The range, it was said, within which the calculation was made was between 1 in 45 billion and 1 in 14 trillion. […] In other words, it would appear to be necessary to search well beyond this planet and conceivably this galaxy to find a match”. This argument leads the layperson to think that DNA is sufficient for individualisation, when it is not [70]). In some countries, for example, the UK [71], it is still current practice to report all LRs that are computed as greater than 10^9^ to be “of the order of one billion”, even though the number of loci in routine use is much greater than the original 10 at the time of the Foreman and Evett study. This seems to cause no problem, but it is a peculiar state of affairs.

In addition, it has been advocated that one should consider the possibility of an error, [72] as DNA analysis is not error free [73]. When we do, the value of the results will be largely dominated by this probability [3,7,74].

### 13.2. With High LRs, Does It Really Matter if One Transposes the Conditional?

Some might advocate that we should not split hairs and that if the LR is very high, then the effect of prior odds is washed away and it is not a real issue if the scientist transposes the conditional. Reporting and testimony represent the end-product of the scientist’s job. Whether or not it would be acceptable in some cases to let the court believe that the results allow the scientist to assign a posterior probability, is a matter of ethics and code of conduct. The scientist’s remit is scientific results and their value in the context of the alleged facts, not the facts themselves. It is the duty of factfinders to consider both the results and the alleged facts.

## 14. Conclusions

Understanding and conveying uncertainty is a difficult endeavour. As we cannot suppress uncertainty, we need to face it and include it in our actions [14]. For DNA scientists, this involves putting measures in place to ensure that results are appropriately interpreted and their value well communicated. Identifying the issue with which forensic science can help and using the framework we presented is the first step: it allows scientists to rationally assess and present the value of their results in a transparent way. It ensures that the LRs assigned given sub-source-level propositions are not carried over to activity-level propositions [75]. It is important to acknowledge that DNA, although extremely useful, is not the magic bullet. There will be situations where DNA comparisons alone will not be helpful. If the person involved has legitimate access to the object or person, and one can safely assume that the DNA comes from this person, then it does not make much sense to consider the probability of the results given the DNA is from an unknown person, as this is not what is alleged. Another example could be a case where the issue is not the person from whom the DNA comes from, but the activities that led to the deposition of the material: if the activities alleged by both parties are very similar to each other, the DNA outcome will not allow discrimination of the propositions. These points need to be conveyed to the trier of facts.

To help communication, harmonisation, and to raise the standards of interpretation, several measures could be taken: (1) invest in the formal education of the key players (i.e., the messenger and the recipient of the information), (2) provide the forensic community with appropriate tools and knowledge, and (3) make interpretation part of the scope of accreditation. Having technical assessors and adequate proficiency tests would allow for the monitoring of the evaluation of DNA results given both sub-source and activity-level propositions. It would ensure DNA scientists adhere to the principles of interpretation as advocated in the Forensic Science Regulator of England and Wales codes of practice and conduct for evaluative opinions [12]. We note also that in some countries, such as the Netherlands, there have been initiatives by The Netherlands Register of Court Experts [76,77] to guarantee and promote the quality of the contribution made by court experts to the legal process. This is an avenue other countries could pursue to ensure that experts are fully qualified and certified for both types of reporting.

## Figures and Tables

**Table 1:** Differences between investigative and evaluative roles.

Investigative Role	Evaluative Role
Tends to be crime-focused.	Tends to be suspect-focused.
Tends to be at the beginning of the criminal justice process.	Tends to be at the culmination of the criminal justice process.
Helps the investigator make decisions.	Helps the court take decisions.
Type of questions: What happened? What is this? Who could be involved?	Type of questions: Is Mr Smith the source of the DNA or is it someone else? Did Mr Smith drive the car or was he a passenger?
Suggests explanations for the observations and gives guidance.	Evaluates the observations (i.e., results) given at least two mutually exclusive propositions.
Open-ended process.	More formal and structured.
Explanations need to encompass all realistic possibilities to avoid bias and avoid potentially misleading the inquiry.	Assigned probabilities should be calibrated; rates of misleading opinions should be low and documented.

**Table 2:** Statements with transposed conditionals and their associated correct statement.

	Incorrect or Ambiguous Statement (A)	Correct Statement (B)	Notation
1.	Given the DNA results, it is a billion times more likely that the DNA is from Ms Jones than from an unknown unrelated person.	The results are a billion times more probable if the DNA is from Ms Jones rather than if it is from an unknown unrelated person.	A: Pr(*H*_p_|*E*,*I*)/Pr(*H*_d_|*E*,*I*) B: Pr(*E*|*H*_p_,*I*)/Pr(*E*|*H*_d_,*I*) Note: In A, the use of the term “that”is always suspicious.
2.	The likelihood ratio calculated the probability that the DNA evidence observed in the profile originated from the applicant, rather than another person chosen at random from the Australian Caucasian population. (Sometimes, scientists will write “the probability that a randomly selected person would have this DNA profile is 1 in a billion.”. However, the alternative source of the DNA is not “selected at random”, but rather an unknown person, related or not to the persons of interest depending on the case).	The likelihood ratio represents the ratio of two probabilities: the probability of the result given that the DNA is from the applicant and the probability of the result given the DNA is from an unknown person from the Australian Caucasian population.	A: Pr(*H*_p_|*E*,*I*)/Pr(*H*_d_|*E*,*I*) B: Pr(*E*|*H*_p_,*I*)/Pr(*E*|*H*_d_,*I*) Note: a word such as « if » or « given » should always be present when using conditional probabilities.
3.	Participants of the proficiency test were asked whether their DNA results more probably originated due to the disputed activity or social interactions. (In the exercise, it read as “Participants were required to consider each DNA profile separately and assess whether they originated either due to primary or secondary transfer” however, it is best not to put the word transfer in the propositions as transfer/persistence and recovery are factors that scientists will take into account in their evaluation.)	Participants of the proficiency test were asked whether their DNA results were more probable given the disputed activity than given social interactions.	A: Pr(*H*_p_|*E*,*I*)/Pr(*H*_d_|*E*,*I*) B: Pr(*E*|*H*_p_,*I*)/Pr(*E*|*H*_d_,*I*) Note: In A, the absence of the term “if [the proposition]”or “given [the proposition]”is a clue that the statement needs review.
4.	The probability that the DNA comes from a random unrelated person is one in a million.	The probability of the results if the DNA comes from a random unrelated person is one in a million.	A: Pr(*H*_d_|*I*) B: Pr(*E*|*H*_d_,*I*) Note: statement B is correct (not transposed) but not balanced since Pr(*E*|*H*_p_,*I*) is missing.
5.	Considering the genetic characteristics of the trace, it is a million times more likely that Mr S is the source of the DNA rather than his uncle.	The genetic characteristics of the trace are a million times more likely if Mr S is the source of the DNA rather than his uncle.	A: Pr(*H*_p_|*E*,*I*)/ Pr(*H*_d_|*E*,*I*) B: Pr(*E*|*H*_p_,*I*)/Pr(*E*|*H*_d_,*I*) Note: in statement A, the phrase “considering the genetic characteristics of the trace” implies that the DNA characteristics represent the conditioning information (thus are present behind the conditional bar). However, the conditioning information should be the propositions and the information; the term “given” should be associated with the propositions and information, not with the results.
6.	As explained by this Court in Tuite [No 1] for each DNA sample where the suspect cannot be excluded as a contributor, a ratio was calculated which shows how much more likely it is that the suspect was the source of the DNA (or a contributor to it) than that some other person chosen at random from the population was the source (or a contributor).	As explained by this Court in Tuite [No 1] for each DNA sample where the suspect cannot be excluded as a contributor, a ratio was calculated which shows how much more likely the DNA results would be given the suspect were the source of the DNA (or a contributor to it) than given an unknown person from the population were the source (or a contributor).	A: LR defined incorrectly as Pr(*H*_p_|*E*,*I*)/ Pr(*H*_d_|*E*,*I*) B: LR is defined as LR = Pr(*E*|*H*_p_,*I*)/ Pr(*E*|*H*_d_,*I*)
7.	The most favoured proposition is that S is the source of the DNA rather than an unknown unrelated person.	The DNA results strongly support the proposition that S, rather than an unknown unrelated person, is the source of the DNA.	Note: in A the use the term favour could be read as saying the first proposition is most likely. The term “support” is more appropriate.

**Table 3:** Posterior probabilities with the same LR of a million, but different prior odds. In the first case, although results support the first proposition, the most favoured proposition is the alternative, with a probability of 91%. When prior odds are equal to the LR, the probability of the proposition is 50%. If prior odds are 1:1, then posterior odds are equal to the LR.

Prior Odds	LR	Posterior Odds	Posterior Probability of the First Proposition	Posterior Probability of the Alternative
1 to 10 million	1 × 10^6^	1 to 10	9%	91%
1 to 1 million	1 × 10^6^	1 to 1	50%	50%
1 to 10,000	1 × 10^6^	100 to 1	99%	1%

**Table 4:** Evaluative examples of pairs of mutually exclusive propositions at different levels in the hierarchy that DNA scientists could contribute to addressing provided they have knowledge that is needed but which otherwise would be unavailable to the court.

Level	Question/Issue	Results	Example of Pairs of Propositions
Source	Is the POI the source of the body fluid?	DNA profiling comparison.	Mr A is the source of the blood. An unknown unrelated person is the source of the blood.
Sub-source	Is the POI the source of the DNA?		Mr A is the source of the DNA. An unknown unrelated person is the source of the DNA.
Sub-sub-source	Is the POI the source of the part of the mixture?		Mr A is the major contributor of the DNA mixture. An unknown unrelated person is the major contributor of the DNA mixture.
Activity	Did the POI do the activity?	Presence/absence of DNA at different locations. Quantity/quality of the DNA. (DNA profiling comparison) *. Presumptive tests. Multiple traces from same activity.	Mr A and Ms B had penile-vaginal intercourse Mr A and Ms B only had social activities as described in the case information. Mr Smith was the driver and Mr Jones the passenger at the relevant time. Mr Jones was the driver and Mr Smith the passenger at the relevant time.
Offence	Is the POI the offender?	Multiple traces possibly resulting from different activities compared to Mr A. Presence/absence of DNA. Quantity/quality of the DNA. (DNA profiling comparison) *. Presumptive tests.	Mr A is the burglar (in the sense of “Mr A did the specified activities that the burglar has allegedly done, as specified in the paragraph on case information”). Mr A has nothing to do with the burglary.

* In some cases, considering whether or not the DNA is from the POI can have an impact on the evaluation.

**Table 5:** Criteria that help spotting whether propositions are formulated in a meaningful way.

Criteria for Propositions	Basis of the Criteria
They come in pairs, so there are at least two propositions.	To ensure a balanced view representing both parties.
They are based on the views of the parties and contextual information.	So that the evaluation is relevant to the case.
They are formal and relate to inductive inference.	To allow scientists logically to assess their findings
They are mutually exclusive.	If not, a LR cannot be used.
They represent the positions that prosecution and defence respectively would be expected to take at court.	So that there is one value of the findings and not several values (one LR, not several).
Propositions are about “causes”, not results.	To enable the scientists to add value and expertise that is needed for the understanding the case.

**Table 6:** Examples of propositions that should be avoided and how to formulate them in a more meaningful way.

	Examples to Avoid	More Meaningful Examples	Comments
1.	Mr Smith was not the passenger.	Mr Smith was the driver.	If propositions are vague, it is difficult to assign a probability, unless this is specified in the paragraph summarising task-pertinent information.
2.	The DNA is from someone other than Mr Smith.	The DNA is from an unknown person.	
3.	Mr Smith was in recent contact with the victim.	Mr Smith visited the victim’s house as described in the case information.	
4.	The matching profile comes from Mr Smith.	The DNA is from Mr Smith.	“Matching profile” is a result: it should not be included in the proposition.
5.	The male DNA is from Mr Smith or someone of his paternal lineage.	The male DNA is from Mr Smith.	Here, it is Mr Smith who would be on trial, not his paternal lineage. If one needs to consider a person from the paternal lineage, this should be done in the alternative. It is the methods that depend on the issue and not the reverse (i.e., propositions do not depend on methods).
6.	Mr A’s DNA was transferred on the drug package via Officer B.	Officer B arrested Mr A before seizing the drug package.	As written, the statement says that “DNA was transferred”. This is essentially an explanation for the observations. The probability of the findings given this explanation is approaching 1. The probability of DNA being transferred needs to be taken into account by the scientist when evaluating results given activity-level propositions and the case information. That transfer has occurred cannot be included in the proposition for evaluation.

**Table 7:** Examples of explanations that can be given as investigative opinions, but not evaluative.

Observations	Explanations
DNA profile of Mr Smith is compatible with the DNA profile of the trace.	The DNA was planted by the police.
	The real offender has the same DNA profile
	His DNA was transported from his beer can to the door by the wind.
	The government synthesised a DNA profile with the same allelic designations as him.
	The DNA is from his lost twin.
	There was contamination.
	The DNA was secondarily transferred.
	The person was recently in contact with the object.
No semen.	There was no ejaculation.
	A condom was used.
	There was no intercourse.
	There was intercourse but all trace of semen was lost following a vaginal douche.
	The swab taken did not recover the material that was present because of bad procedures.

**Table 8:** Examples of useful caveats in written statements.

Reason for the Caveat	Example
Underline the importance of case information and propositions.	The evaluation presented in this report is crucially dependent on the information provided to the examiner and on the propositions addressed. Any change in the framework of circumstances or in either of the propositions should be seen as sufficient reason for a new evaluation. If some information were found to be incorrect, or if new information were made available, I would need to re-evaluate the value of the findings. Re-evaluation will be more effective if performed in advance of a trial.
Explain what a LR is and is not.	A likelihood ratio indicates if and to what extent the DNA analysis results support one proposition over another. It is not possible, on this basis alone, to determine which is the most probable proposition. To assign the probability of a proposition, the DNA analysis results should be combined with other information in the case. This is generally not considered to be the remit of the DNA scientist.
Alert on the difference between source and activity issues.	This report does not provide any information on the mechanisms or actions that led to the deposition of the recovered biological material. It only provides information regarding its origin (e.g., who is the source of the DNA). Should there be any issue regarding the transfer mechanisms that led to the detection of this material, the results should be evaluated given the alleged activities.
Delineate the meaning of a verbal scale.	The likelihood ratio is a numerical value. Words can be assigned to brackets of numerical values and used as a descriptor of the results’ support for a proposition. Several verbal equivalence tables have been published, and it is above all a convention.

## Data Availability

Not applicable.
